# Genomic expression profiling and bioinformatics analysis of pancreatic cancer

**DOI:** 10.3892/mmr.2015.3917

**Published:** 2015-06-11

**Authors:** DONG-YAN HAN, DA FU, HAO XI, QIAN-YU LI, LI-JIN FENG, WEI ZHANG, GUO JI, JIA-CHENG XIAO, QING WEI

**Affiliations:** Department of Pathology, Shanghai Tenth People's Hospital, Shanghai 200072, P.R. China

**Keywords:** pancreatic cancer, gene expression profiling, DNA microarray, type I diabetes mellitus, systemic lupus erythrmatosus

## Abstract

Pancreatic cancer is a polygenic disease and the fourth leading cause of cancer-associated mortality worldwide; however, the tumorigenesis of pancreatic cancer remains poorly understood. Research at a molecular level, which includes the exploration of biomarkers for early diagnosis and specific targets for therapy, may effectively aid in the diagnosis of pancreatic cancer in its early stages and in the development of targeted molecular-biological approaches for treatment, thus improving prognosis. By conducting expression profiling in para-carcinoma, carcinoma and relapse of human pancreatic tissues, 319 genes or transcripts with differential expression levels >3-fold between these tissue types were identified. Further analysis with Gene Ontology and the Kyoto Encyclopedia of Genes and Genomes demonstrated that the translation, nucleus assembly processes and molecular functions associated with vitamin B6 and pyridoxal phosphate binding in pancreatic carcinoma were abnormal. Pancreatic cancer was additionally identified to be closely associated with certain autoimmune diseases, including type I diabetes mellitus and systemic lupus erythematosus.

## Introduction

Pancreatic cancer is globally the fourth leading cause of cancer-associated mortality for men and women, based on incidence and mortality statistics. There were 337,872 novel pancreatic cancer cases reported and 330,372 cases of pancreatic cancer-associated mortality in 2012, accounting for 2.4% of the annual novel cancer cases in 2012 and ranking as the 12th most prevalent cancer world wide (Globocan 2012; http://globocan.iarc.fr/Pages/fact_sheets_population.aspx). The average life expectancy upon diagnosis is between four and eight months, and individuals undergoing surgery to remove the carcinoma have an ~30% five-year survival rate (1). However, due to late diagnosis, only 10% of diagnosed patients are eligible for potentially curative surgery (1). Due to the fact that the causes of pancreatic cancer remain to be fully elucidated and no specific symptoms have been identified for early-stage diagnosis, pancreatic cancer remains difficult to diagnose.

Pancreatic cancer is a polygenic disease, as are the majority of cancer types (2, 3). The accumulation of multiple genetic defects has an effect on tumorigenesis (4). The arrival and advancement of DNA microarray technology make it possible to monitor the expression levels of a vast number of genes or transcripts in a single microchip. Thus, microarray technology has become a key tool in the investigation of key genes associated with the progression of this malignancy. Gene expression profiling, which is based on DNA microarray technology, has allowed for the identification of hundreds of genes with differential expression in pancreatic carcinoma (5). Genes with the most up/downregulated expression levels in pancreatic carcinoma are p16, p53, K-ras and Smad4, as previously reported (6). These genes are suggested to serve as predictive biomarkers for early diagnosis. Bioinformatics analysis allows for the mapping of genes with differential expression levels to metabolic or signalling pathways, which may provide potential targets for the design of novel anti-cancer drugs (7). The Ras signaling pathway, for example, has attracted attention as an anti-cancer drug target, due to its important function in tumorigenesis (8). For pancreatic cancer, Wnt (9), Notch (10) and Hedgehog (11) pathways have been additionally identified as being of marked significance.

Given the complexity of the genome, it is suggested that numerous genes associated with pancreatic cancer have remained to be identified. Thus, the present study aimed to investigate and enhance the understanding of the underlying molecular mechanisms of pancreatic cancer by undertaking gene expression profiling on a pancreatic carcinoma sample in Shanghai, China. Human whole genome microarray analysis was used to identify the differentially expressed genes between para-carcinoma, carcinoma and relapse human pancreatic cancer tissues.

## Materials and methods

### Tissue samples

The para-carcinoma, carcinoma and relapse pancreatic carcinoma tissues were obtained from a patient (46 years old, female, stage II) undergoing cancer resection at Shanghai Tenth People's Hospital (Shanghai, China). Written informed consent was obtained from the patient and ethical approval of the present study was obtained from the ethical committee of Shanghai Tenth People's Hospital (Shanghai, China).

### RNA extraction

RNA samples from matched para-carcinoma, carcinoma and relapse pancreatic carcinoma tissues were extracted using TRIzol reagent (Invitrogen Life Technologies, Carlsbad, CA, USA). A total of 1 ml TRIzol was used for every 100 mg tissue. Total RNA was isolated using phenol/chloroform (Sinopharm Chemical Reagent Co., Ltd, Shanghai, China) according to the manufacturer's instructions. Subsequent to the precipitation of RNA, 75% (v/v) ethanol was used to wash out the salts. The RNA was then air-dried and dissolved in RNase-free water. The quality and quantity of total RNA was determined using a NanoDrop 2000 (Thermo Fisher Scientific, Waltham, MA, USA).

### Microarray assay

The Agilent Microarray Platform (Agilent Technologies, Inc., Santa Clara, CA, USA) was used to conduct the microarray analysis. Sample preparation and the follow-up hybridization were performed according to the manufacturer's instructions. Total RNA (1 *µ*g) was extracted from each sample as mentioned above, and the Agilent Quick Amp Labeling kit (protocol version 5.7; Agilent Technologies, Inc.) was used to amplify and transcribe the RNA into fluorescent cRNA following the manufacturer's instructions. Sample labeling was performed using the Agilent Quick Amp Labeling kit, while subsequent hybridization was performed in SureHyb Hybridization Chambers (Agilent Technologies, Inc.). The labelled cRNA was then hybridized onto the Whole Human Genome Oligo Microarray (4×44 K; Agilent Technologies, Inc.). Arrays were scanned with the G2505B Scanner (Agilent Technologies, Inc.) subsequent to washing of the slides.

### Data analysis

The acquired array images were analyzed with Agilent Feature Extraction software, version 10.7.3.1, while GeneSpring GX software, version 11.5.1 (Agilent Technologies, Inc.) was used for quantile normalization and data processing.

Among the 45,000 genes or transcripts included in the microarray, 7,937 genes or transcripts with valid values detected in all three groups measured (carcinoma, para-carcinoma and relapse tissues) were used for the subsequent analysis. Genes with differential expression levels in different tissues were identified by fold-change filtering. Expression levels of genes were normalized by log2 transformation for the subsequent analysis. Pairwise comparisons were completed between the expression levels of the same gene or transcript in any two tissues. Genes or transcripts exhibiting fold-changes >1.5- and 3-fold in expression levels in a minimum of one pairwise comparison were selected for further analysis, and bioinformatics analysis was conducted on the genes or transcripts with alterations in expression levels of >3-fold.

Analysis results from Gene Ontology (GO) and Kyoto Encyclopedia of Gene and Genomes (KEGG; http://www.genome.jp/kegg/) databases were gathered and enriched by using the online Database for Annotation, Visualization and Integrated Discovery server (DAVID; http://david.abcc.ncifcrf.gov/) with the standard enrichment computation method (12).

## Results

### Microarray analysis

**Figure 1:**
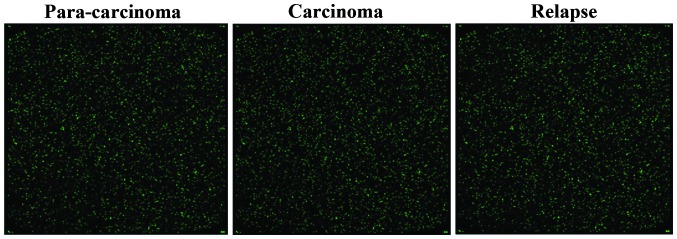
Gene chip hybridization fluorescence signal image in para-carcinoma, carcinoma and relapse tissues of human pancreatic cancer.

The microarray assay was qualified according to quality standards, the experimental systems were observed to be stable and the fluorescent signal intensity was strong and homogenous (Fig. 1). cRNAs were hybridized onto the Whole Human Genome Oligo Microarray and 7,937 probes exhibited clear signals in all three chips simultaneously, representing 17.63% of the 45,000 probes assessed. Subsequent to differential expression level analysis, genes or transcripts corresponding to 3,298/7,937 probes were observed to exhibit alterations in expression levels of >1.5-fold. Among these, 319 genes or transcripts were observed to have a fold change of ≥3-fold.

### Gene ontology analysis

A total of 319 genes or transcripts associated with pancreatic cancer were observed to exhibit a ≥3-fold change in expression levels in the present study. Subsequently, the GO database was used to analyze these genes and DAVID was used for the enrichment terms.

A total of 23 functional description nodes were identified to be associated with biological processes, with P<0.01 (Table I). According to their P-values (low-high), the top five terms were: Translational elongation, translation, nucleosome assembly, chromatin assembly and protein-DNA complex assembly. All of these terms were associated with cell metabolic processes. In addition, terms which are involved in immune response and metal ion metabolic processes were observed.

Furthermore, 26 functional description nodes were identified to be associated with cellular components, with P<0.01 (Table II). The top five terms were identified to be: Cytosolic ribosome, ribosomal subunit, cytosolic small ribosomal subunit, cytosolic part and small ribosomal subunit.

Finally, 7 functional description nodes were identified to be associated with molecular function, with P<0.01. These nodes were: Structural constituent of ribosome, structural molecule activity, protein binding, pyridoxal phosphate binding, vitamin B6 binding, binding and cadmium ion binding (Table III).

### KEGG pathway analysis

**Figure 2:**
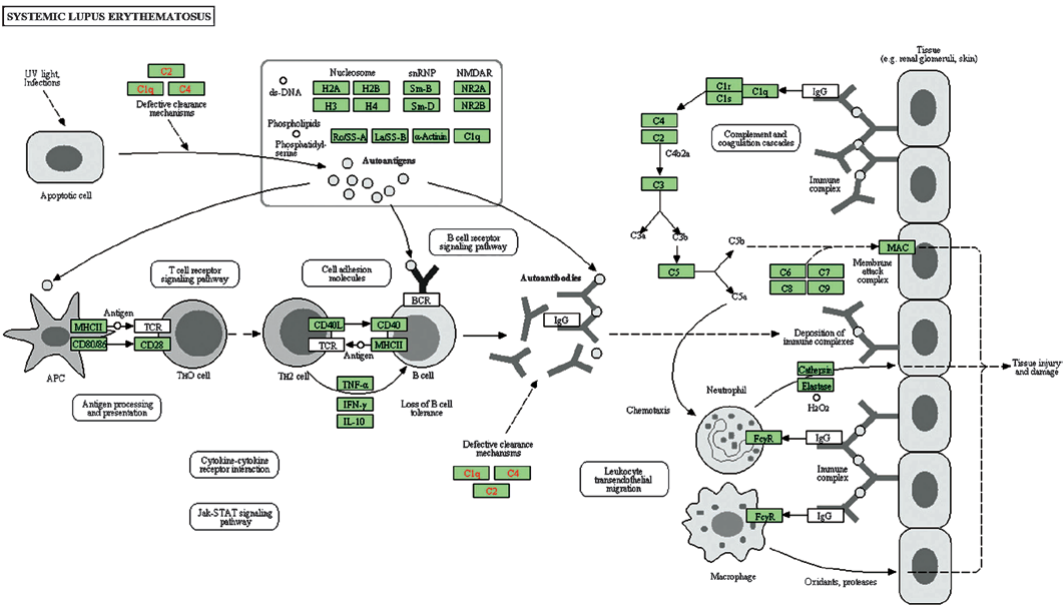
Schematic of the systemic lupus erythematosus pathway in which HLA-DPB1, HIST1H4J, HIST1H2BO, H3F3C, H2AFY, H3F3A, HIST2H3D, HIST1H4D, HIST2H4B, HIST1H2BL and HIST1H4K are involved. IgG, immunoglobulin G; UV, ultraviolet; TCR, T-cell receptor; BCR, B-cell receptor; Fcyr, Fc receptor for IgG.

**Figure 3:**
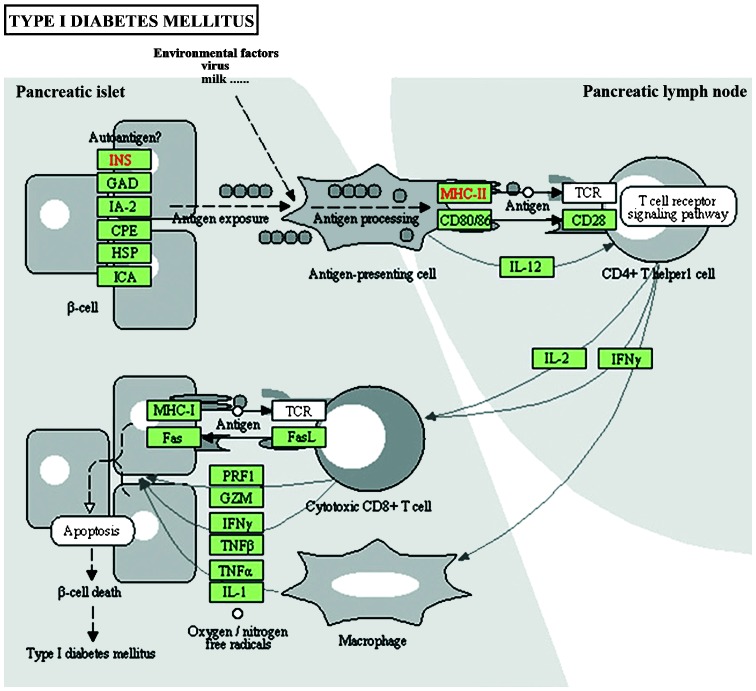
Schematic of the type I diabetes mellitus pathway in which HLA-A, HLA-B, HLA-C, HLA-DPB1 and GAD1 are involved.

KEGG pathway analysis was conducted on genes with an alteration in expression levels of >3-fold between the carcinoma, para-carcinoma and relapse human pancreatic cancer tissues. Of these, the genes associated with systemic lupus erythematosus (SLE) and type I diabetes mellitus are presented (Table IV). In systemic lupus erythematosus, 11 key genes were differentially expressed between carcinoma, para-carcinoma and relapse tissue, including: HLA-DPB1, HIST1H4J, HIST1H2BO, H3F3C, H2AFY, H3F3A, HIST2H3D, HIST1H4D, HIST2H4B, HIST1H2BL and HIST1H4K (Fig. 2, Table V). In type I diabetes mellitus, HLA-A, HLA-B, HLA-C, HLA-DPB1 and GAD1 (Fig. 3, Table VI) exhibited significantly different expression levels.

## Discussion

Pancreatic cancer is a lethal malignancy with few effective therapies currently available (13). It is the fourth leading cause of cancer-associated mortality, with an overall five-year survival rate of <5% (14), which has remained unaltered for 50 years. With the availability of DNA microarray and next generation sequencing, it is now possible to study diseases, including various types of cancer, at the 'omic' level (15). DNA microarray gene expression profiling has previously been successfully applied in large-scale analyses of differentially expressed genes involved in tumorigenesis (16). Gene expression profiling has previously been used in numerous studies focusing on pancreatic cancer. Chang *et al* (17) demonstrated that 3,853 genes displayed differential expression by >1.5-fold in pancreatic carcinoma tissue. Of these genes, the expression levels of 2,512 genes were upregulated and 1,341 genes were downregulated. Nakamura *et al* (18) identified 260 upregulated and 346 downregulated genes involved in pancreatic cancer.

In the present study, the gene expression levels between carcinoma, relapse carcinoma and para-carcinoma of human pancreatic cancer tissues were compared. Differentially expressed genes were observed and analyzed using GO term and KEGG pathway enrichment analysis.

Using GO term analysis, differentially expressed genes were observed in the present study, which were identified to be involved in biological processes and associated with translation, the nucleus and chromatin assembly. This is consistent with the knowledge that the nuclei in carcinoma cells are misshapen and enlarged (19). In the cellular component domain, the majority of the enriched terms were associated with the ribosomes. In the GO analysis domain of molecular function, terms regarding the structural constitution of ribosomes and protein binding were highlighted. Of note, differentially expressed genes identified to be associated with molecular function included terms of pyridoxal phosphate (PLP) and vitamin B6 binding, and PLP is the active form of vitamin B6 (20). Johansson *et al* (21) reported that the serum vitamin B6 levels were inversely associated with the risk of lung cancer and Wu *et al* (22) demonstrated that serum PLP levels were inversely associated with the risk of breast cancer. Overall, this suggested that the genes associated with vitamin B6 binding are involved in tumorigenesis.

Using KEGG analysis, the pathways of SLE and type I diabetes mellitus were identified to be significantly associated with pancreatic cancer. SLE is a systemic autoimmune disease, which can affect any part of the body (23). At present, it is accepted that SLE is associated with an increased risk of certain types of cancer. Previous studies have demonstrated the association between SLE and non-Hodgkin lymphoma (NHL) (24–29) as well as Hodgkin lymphoma (30,31). The risk of NHL was found to be increased by several fold in a SLE population, compared with that of a healthy population (32). Increased risks of breast (29), lung (25,33–37), cervical (26,29) and endometrial cancer (38) in patients with SLE have been observed by cohort studies. Type I diabetes mellitus results from the autoimmune destruction of the insulin-producing cells in the pancreas (39). By meta-analysis, Stevens *et al* (4 0) identified an increased risk of pancreatic cancer in a population with type I diabetes mellitus. A population-based cohort study in Sweden conducted by Zendehdel *et al* (41) demonstrated that patients with type I diabetes mellitus additionally exhibited increased incidences of stomach, cervical and endometrial cancer (41).

In conclusion, the present study suggested that the abnormal expression levels of multiple genes contribute to the incidence of pancreatic cancer. Additional diseases, including type I diabetes and SLE, are closely associated with the tumorigenesis of pancreatic carcinoma. Although the specific functions of these genes with differential expression levels and their mechanisms require further investigation, the results of the present study may aid clinicians in the early diagnosis of pancreatic cancer and in the production of novel targeted therapies.

## Figures and Tables

**Table I:** Gene ontology analysis of differentially expressed genes associated with biological processes.

Term	Gene count (n)	%^a^	P-value
Translational elongation	16	7.2398190	9.70×10^−13^
Translation	18	8.1447964	4.72×10^−7^
Nucleosome assembly	8	3.6199095	6.50×10^−5^
Chromatin assembly	8	3.6199095	8.14×10^−5^
Protein-DNA complex assembly	8	3.6199095	1.08×10^−4^
Nucleosome organization	8	3.6199095	1.24×10^−4^
Response to metal ion	9	4.0723982	1.60×10^−4^
Antigen processing and presentation of peptide antigen	5	2.2624434	3.24×10^−4^
Antigen processing and presentation	7	3.1674208	4.75×10^−4^
DNA packaging	8	3.6199095	5.12×10^−4^
Response to stimulus	62	28.054299	6.66×10^−4^
Response to inorganic substance	10	4.5248869	8.13×10^−4^
Chromatin assembly or disassembly	8	3.6199095	8.35×10^−4^
Antigen processing and presentation of peptide antigen via MHC class I	4	1.8099548	0.00101320
Immune response	19	8.5972851	0.00156179
Negative regulation of apoptosis	12	5.4298643	0.00359381
Negative regulation of programmed cell death	12	5.4298643	0.00399353
Negative regulation of cell death	12	5.4298643	0.00407759
Response to calcium ion	5	2.2624434	0.00420590
Iron ion transport	4	1.8099548	0.00490087
Regulation of calcium ion transport into cytosol	4	1.8099548	0.00490087
Response to chemical stimulus	27	12.217195	0.00526131
Ribosomal small subunit biogenesis	3	1.3574661	0.00730011

a Percentage of the counted genes among all the genes with a >3-fold change in expression level. MHC, major histocompatibility complex.

**Table II:** Gene ontology analysis of differentially expressed genes associated with cellular components.

Term	Gene count (n)	%^a^	P-value
Cytosolic ribosome	18	0.0814480	2.07×10^−17^
Ribosomal subunit	18	0.0814480	6.53×10^−14^
Cytosolic small ribosomal subunit	12	0.0542986	4.40×10^−13^
Cytosolic part	18	0.0814480	1.16×10^−12^
Small ribosomal subunit	13	0.0588235	4.31×10^−12^
Ribosome	19	0.0859729	3.42×10^−11^
Ribonucleoprotein complex	23	0.1040724	7.30×10^−8^
Macromolecular complex	63	0.2850679	1.83×10^−6^
Nucleosome	8	0.0361991	6.37×10^−6^
Cytosol	34	0.1538462	1.09×10^−5^
Protein-DNA complex	8	0.0361991	4.98×10^−5^
Cytoplasmic part	79	0.3574661	1.53×10^−4^
Cytoplasm	107	0.4841629	1.81×10^−4^
Non-membrane-bounded organelle	49	0.2217195	2.04×10^−4^
Intracellular non-membrane-bounded organelle	49	0.2217195	2.04×10^−4^
MHC class I protein complex	5	0.0226244	2.52×10^−4^
Intracellular part	141	0.6380090	3.21×10^−4^
MHC protein complex	6	0.0271493	4.34×10^−4^
Intracellular	144	0.6515837	4.91×10^−4^
Cytosolic large ribosomal subunit	5	0.0226244	8.31×10^−4^
Organelle part	68	0.3076923	8.84×10^−4^
Intracellular organelle	122	0.5520362	0.0010071
Organelle	122	0.5520362	0.0010775
Intracellular organelle part	67	0.3031674	0.0012716
Chromatin	9	0.0407240	0.0018679
Large ribosomal subunit	5	0.0226244	0.0067159

a Percentage of the counted genes among all the genes with a >3-fold change in expression levels. MHC, major histocompatibility complex.

**Table III:** Gene ontology analysis of differentially expressed genes associated with molecular function.

Term	Gene count (n)	%^a^	P-value
Structural constituent of ribosome	17	0.0769231	8.56×10^−11^
Structural molecule activity	21	0.0950226	3.80×10^−5^
Protein binding	118	0.5339367	1.44×10^−4^
Pyridoxal phosphate binding	5	0.0226244	0.0033357
Vitamin B6 binding	5	0.0226244	0.0033357
Binding	157	0.7104072	0.0041273
Cadmium ion binding	3	0.0135747	0.0054982

a Percentage of the counted genes among all the genes with a >3-fold change in expression levels.

**Table IV:** KEGG pathway analysis results.

KEGG pathway ID	Pathway name	Gene count (n)	Percentage of counted genes	NCBI gene ID	Fold enrichment	P-value
hsa05322	Systemic lupus erythematosus	11	4.977%	3115, 8363, 8348, 440093, 9555, 3020, 653604, 8360, 554313, 8340, 8362	4.565656	0.001602
hsa04940	Type I diabetes mellitus	5	2.262%	2571, 3115, 3107, 3105, 3106	5.380952	0.036427

KEGG, Kyoto Encyclopedia of Genes and Genomes; NCBI, National Center for Biotechnology Information; hsa, *Homo sapiens*.

**Table V:** Genes with >3-fold change in expression levels in pancreatic cancer associated with systemic lupus erythematosus.

Gene ID	Gene symbol	fcCP	fcRP	fcCR	Description
3115	HLA-DPB1	0.603255	1.960175	0.307756	Major histocompatibility complex, class II, DP beta 1
8363	HIST1H4J	0.308301	0.880094	0.350304	Histone cluster 1, h4j
8348	HIST1H2BO	0.368137	1.208018	0.304744	Histone cluster 1, h2bo
440093	H3F3C	0.719753	3.972665	0.181176	H3 histone, family 3C
9555	H2AFY	1.356658	0.260470	5.208496	H2A histone family, member Y
3020	H3F3A	0.298552	0.615353	0.485173	H3 histone, family 3A
653604	HIST2H3D	0.296694	0.722873	0.410437	Histone cluster 2, h3d
8360	HIST1H4D	0.421040	1.396684	0.301457	Histone cluster 1, h4d
554313	HIST2H4B	0.333331	0.730559	0.456268	Histone cluster 2, h4b
8340	HIST1H2BL	0.224496	0.710622	0.315914	Histone cluster 1, h2bl
8362	HIST1H4K	0.335573	1.021847	0.328399	Histone cluster 1, h4k

fcCP, fold change of expression levels of genes in carcinoma tissue compared with that in para-carcinoma tissue; fcRP, fold change of expression levels of genes in relapse tissue compared with that in para-carcinoma tissue; fcCR, fold change of expression levels of genes in carcinoma tissue compared with that in relapse tissue.

**Table VI:** Genes with >3-fold change in expression levels in pancreatic cancer associated with type I diabetes mellitus.

Gene ID	Gene symbol	fcCP	fcRP	fcCR	Description
2571	GAD1	1.062045	4.264469	0.249045	Glutamate decarboxylase 1
3115	HLA-DPB1	0.603255	1.960175	0.307756	Major histocompatibility complex, class II, DP beta 1
3105	HLA-A	0.312182	0.491002	0.635806	Major histocompatibility complex, class I, A
3106	HLA-B	0.299524	0.644979	0.464393	Major histocompatibility complex, class I, B
3107	HLA-C	0.330177	0.661858	0.498863	Major histocompatibility complex, class I, C

fcCP, fold change of expression levels of genes in carcinoma tissue compared with that in para-carcinoma tissue; fcRP, fold change of expression levels of genes in relapse tissue compared with that in para-carcinoma tissue; fcCR, fold change of expression levels of genes in carcinoma tissue compared with that in relapse tissue.
